# What is dyslexia? An exploration of the relationship between teachers' understandings of dyslexia and their training experiences

**DOI:** 10.1002/dys.1593

**Published:** 2018-07-17

**Authors:** Cathryn Knight

**Affiliations:** ^1^ School of Social Sciences Cardiff University Cardiff UK

**Keywords:** dyslexia, learning, teaching, training

## Abstract

Given that an estimated 5–10% of the worldwide population is said to have dyslexia, it is of great importance that teachers have an accurate understanding of what dyslexia is and how it effects their students. Using results from a large‐scale survey of teachers in England and in Wales (*N* ≈ 2,600), this paper demonstrates that teachers held a basic understanding of dyslexia, based on the behavioural issues that it is associated with. Teachers lacked the knowledge of the biological (i.e., neurological) and cognitive (i.e., processing) aspects of dyslexia. Moreover, a number of teachers mentioned visual factors in their description of dyslexia, despite there being inconclusive evidence to suggest a direct relationship between visual functioning and dyslexia. Further findings demonstrate the importance of good‐quality teacher training in increasing teachers' confidence working with those with dyslexia, while increasing their knowledge of the cognitive aspects of dyslexia. This paper argues that evidence‐based teacher training, which informs teachers of the up‐to‐date research on the biological, cognitive, and behavioural aspects of dyslexia, is essential to combat misconceptions and ensure that teachers have more nuanced and informed understandings of dyslexia.

**Practitioner points**

- An online survey was completed by teachers in England and Wales (*N* ≈ 2,600).
- When asked to provide a description of dyslexia, a majority (79.5%) used behavioural descriptors, compared with biological descriptors (9%) and cognitive descriptors (39.3%).
- A total of 16.8% of teachers mentioned visual factors in their description of dyslexia, despite this being inconclusive in its relationship with dyslexia.
- The majority of teachers (71.8%) said that dyslexia was “not covered well at all” on their initial teacher training course.
- Those that had additional training were more likely to feel confident working with students with dyslexia and had increased knowledge of the cognitive aspects of dyslexia.

## 1 INTRODUCTION

Dyslexia is a common learning difficulty. Prevalence rates of dyslexia vary depending on how dyslexia is defined. Reported rates range from of 4% to 20% (Butterworth & Kovas, 2013; Coles, 1999; Shaywitz, 1996, 2005; Siegel, 2006; Snowling, 2010). As the average primary school class in the United Kingdom consists of 27.1 students and the average secondary school class is of 20.4 students (Department for Education, 2016), it can be estimated that teachers will have between one and five dyslexic students in each class. In order for teachers to be able to help these students most effectively, it is vital that they understand what dyslexia is and implement the best methods to help these students.

The Rose Report (Rose, 2009) was an independent report, commissioned by the U.K. government, to make suggestions on identifying and teaching children and young people with dyslexia and literacy difficulties. The report calls for all teachers to have a working knowledge of dyslexia. However, adults and young people with dyslexia report that accessing help at school is difficult and “a lack of understanding of the nature of dyslexia leads to unhelpful and damaging comments from some teachers which have long lasting detrimental effects” (Dyslexia Action, 2012, p. 7). This suggests that dyslexia is not adequately understood and dealt with by some schools and teachers. Therefore, the present study investigates what teachers understand about dyslexia and what influences this understanding.

### 1.1 Defining dyslexia

The Dyslexia Action Report advocates that “dyslexia is now clearly ‘on the map’ [and] there is no longer controversy about whether is exists and how to define it” (Dyslexia Action, 2012, p. 7). However, some academics disagree; Elliott and Grigorenko (2014) claim that “the field has been unable to produce a universally accepted definition [of dyslexia] that is not imprecise, amorphous or difficult to operationalise” (p. 5). A research report, commissioned by the Welsh Government, analysed 11 definitions of dyslexia from various organizations. They found that “literacy difficulty is the only universally recognised component of dyslexia [in all definitions]” (Caravolas, Kirby, Fawcett, & Glendenning, 2012, p. 47). This suggests disagreement among organizations about how dyslexia is defined. Brown Waesche, Schatschneider, Maner, Ahmed, and Wagner (2011) state that “an agreed‐on definition is essential for best practice” (p. 296) as, without an agreed definition, an operationalizable understanding of the nature, causes, and best treatments of dyslexia is hard to come by. Therefore, it is of interest to know how those working with dyslexic individuals understand what it is and how this may affect their practice.

The earlier work of Morton and Frith (1995) proposes a causal model of developmental psychopathology. Frith (1995) uses this model in order to address the aforementioned problems in defining and explaining dyslexia. In Frith's causal model framework, dyslexia can be explored through three different levels: biological, cognitive, and behavioural. The model also recognizes the role of the environment and culture in interacting with these three levels. The model aims to explore and explain the differing perspectives of dyslexia by addressing previous paradoxes in dyslexia definitions. The model Frith proposes combines the three levels of dyslexia allowing the perceived paradoxes in definitions to disappear. Frith states that “for a full understanding of dyslexia we need to link together the three levels and consider the impact of cultural factors which can aggravate or ameliorate the condition” (Frith, 1999, p. 211). Therefore, when considering dyslexia, Frith would argue that it is important to understand the biological, cognitive, and behavioural factors. Dyslexia can be viewed as a complex causal chain from biology, to cognition to behaviour. Consequently, it is relevant to argue that all three levels of explanation should be combined for a full understanding of dyslexia.

### 1.2 Dyslexia in policy and practice

Currently children with dyslexia in England are likely to be categorized as having special educational need and disability (SEND). A child with SEND is defined in the Children and Families Act (2014) as

A child of compulsory school age or a young person has a learning difficulty or disability if he or she—

(a) has a significantly greater difficulty in learning than the majority of others of the same age, or

(b) has a disability which prevents or hinders him or her from making use of facilities of a kind generally provided for others of the same age in mainstream schools or mainstream post‐16 institutions. (p. 19)

Wales uses similar criteria based on the definition provided in the Education Act (1994) and defines dyslexia as an additional learning need (ALN). It is easy to see how dyslexia fits into this inclusive category of SEND or ALN. However, there is no doubt that children with many differing issues will also fit into this category. Within this approach to learning difficulties, dyslexia is seen as part of a “continuum of special needs” (Riddick, 2001, p. 223). Therefore, an individual does not need a specific diagnosis of dyslexia to be identified as SEND or ALN and receive the extra help that comes with this. However, in a survey of parents with children with dyslexia, 55% said that their child's teacher failed to notice a problem with their child's development (Dyslexia Action, 2012), suggesting that a diagnosis of dyslexia may be helpful in order to access support.

The Rose Report (Rose, 2009) recognizes this but identifies that it would be impractical and misleading to test all children for dyslexia at school entry. Therefore, Rose calls for educators to “closely observe and assess [children's] responses to pre‐ and early reading activities in comparison to their typically developing peers” (Rose, 2009, p. 11). If teachers do not have a thorough, working understanding of dyslexia, these differences may go unnoticed. Therefore, it is necessary to question what teachers understand dyslexia to be, as this will influence whether dyslexia is picked up and, consequentially, whether the child is able to access the additional support they may need.

### 1.3 Teacher training

Although it is not a teacher's job to diagnose dyslexia, it is important that they have an accurate understanding of the underlying behavioural and cognitive difficulties associated with dyslexia so as to identify those that could be at risk and to intervene appropriately. Research into methods to improve the symptoms associated with dyslexia has shown a positive impact of interventions on the dyslexic individual (Duff & Clarke, 2011; Fletcher, Lyon, Fuchs, & Barnes, 2006; Savage & Carless, 2008; Snowling & Hulme, 2011). The Rose Report (Rose, 2009) used research evidence to conclude that interventions that prioritize phonological skills are effective for teaching reading to children with dyslexia. Phonological processing skills refer to the skills needed to use phonemes (i.e., the sounds in language) to process spoken and written language (Wagner & Torgesen, 1987). The broad category of phonological processing includes the cognitive skills of phonological awareness (the ability of focus on and manipulate the sounds in spoken words) and phonological working memory retrieval (the ability to store and recall the correct phoneme sound from memory). Deficits in these skills are commonly associated with dyslexia. Consequently, as intervening at this level can improve a pupil's literacy performance, it could be argued that teachers need to be trained to understand how to recognize a child who is struggling with these cognitive skills and how to intervene to improve performance. Furthermore, Snowling (2012) states that “a good starting point for developing an intervention is understanding the causes of a disorder” (p. 12). Therefore, it is vital that teachers have a good understanding of both the causes of dyslexia and the evidence‐based interventions that have been proven to benefit those with dyslexia. With this knowledge, teachers will be able to help their students effectively.

The National Teaching Standards framework states that teachers must “have a clear understanding of the needs of all pupils, including those with special educational needs […] and be able to use and evaluate distinctive teaching approaches to engage and support them” (Department for Education, 2011, p. 12). This suggests that every teacher should have the skillset to address individual pupil's needs and respond to these appropriately. However, inadequate teacher training may leave teachers ill‐equipped to meet this requirement.

Research conducted in 1996 suggested that despite the increasing contact that teachers had with pupils with special educational needs (SEN) at the time, it was not adequately covered in initial teacher training (ITT; Garner, 1996). With continuous research into SEN and dyslexia, it would be expected that this situation has improved. However, Webster and Blatchford (2015) conducted qualitative interviews with teachers and teaching assistants and found that over a third of all participants said that they had not received the training they needed to support the students with SEN in their classes. This could be explained by evidence given by the British Dyslexia Association for the Carter Review of ITT, which depicted a “lack of coverage in ITT on dyslexia” (Department for Education, 2015, p. 58). A similar independent report on ITT in Wales states that SEN is “difficult to tackle in sufficient breadth and depth in ITT alone” (Department for Education and Skills, 2013, p. 24). Both reports suggest the use of continued professional development (CPD) following ITT for teachers to gain a better knowledge of the subject. However, Webster and Blatchford's (2015) results suggest that this may not be happening. Consequentially, it is important to know whether teachers believed their ITT covered dyslexia sufficiently and whether they have received any CPD training in addition to their ITT. The impact of good‐quality ITT and additional training can then be explored.

### 1.4 Understanding of dyslexia

A wide range of research has investigated what people understand about dyslexia. In relation to public conceptualizations of dyslexia, Furnham (2013) surveyed 380 participants who he described as the “lay public.” Participants described dyslexia as a “learning disability characterized by problems with words and language”; however, they were unsure about the neurobiological aspects of dyslexia (Furnham, 2013, p. 247). Accordingly, the participants not only showed “modest understanding” of the behavioural nature of dyslexia but also demonstrated naivety about the multifaceted aspects of dyslexia. Applying Furnham's findings to Frith's (1995) model of dyslexia suggests that although the participants showed some awareness of the behavioural aspects of dyslexia, they were ill‐informed about the biological and cognitive characteristics.

To examine students' understanding of dyslexia, Mortimore (2013) surveyed 35 education students. Participants were asked to provide their own definition of dyslexia. All the definitions that participants provided focussed on the difficulties associated with dyslexia, and no strengths associated with dyslexia were mentioned. Additionally, 74.3% of the definitions described the behavioural issues of writing and spelling, whereas 48.6% described reading difficulties. A larger sample of students (*n* = 247) were asked to select the traits most commonly linked with dyslexia. Over 90% of participants endorsed traits of literacy difficulties, indicating a strong preference for students to attribute their understandings of dyslexia as behavioural.

A further study conducted by Bell, McPhillips, and Doveston (2011) compared how teachers in the United Kingdom and Ireland conceptualize dyslexia using Frith's (1995) causal model to map their data. They found that the majority of mainstream teachers used behavioural definitions when asked “how do you define dyslexia?” More than half of the teachers in the United Kingdom did not mention the underlying behavioural and cognitive difficulties associated with dyslexia. When the mainstream teachers were probed further about particular areas of difficulty, results indicated that the teachers did not prioritize the “phonological awareness deficit” and were more likely to mention memory difficulties. This is concerning as, as mentioned previously, a large body of research into the underlying causes of dyslexia contends that phonological awareness is a prerequisite to reading difficulties (Stuart, 2005). Consequently, despite the much earlier body of work put forward by Frith, suggesting that acknowledgement of all three levels is necessary for a good understanding of dyslexia, it would appear that there is a strong tendency to attribute dyslexia to the singular category of the behavioural level.

However, some studies have reported a more holistic understanding of dyslexia in the teaching profession. For example, Regan and Woods (2000) conducted focus groups with 36 teachers and learning support assistants in the United Kingdom and asked them to provide a definition of dyslexia. The focus group participants touched upon all the levels recognized by Frith (1995) by providing biological and cognitive definitions to explain behavioural symptoms. However, the researchers noted that understanding between individuals was varied. Due to the small number of participants in this study, it is necessary to further investigate whether teachers define dyslexia across all three levels, when they are asked individually to provide a definition of dyslexia.

Moreover, Washburn, Binks‐Cantrell, and Joshi (2013) surveyed 171 preservice teachers in the United States and the United Kingdom to investigate whether they held misconceptions about dyslexia. They found that teachers in both countries reported several misconceptions about dyslexia. Most notably, a majority of preservice teachers surveyed stated that dyslexia is caused by issues with visual perception. In particular, this relates to the concept of “visual stress” whereby a person may see a page differently due to distortions of print on a white background. Visual stress has been reported to cause reading fatigue; however, the symptoms can be somewhat overcome by the use of coloured overlays (Wilkins, 2003). Singleton and Trotter's (2005) research on visual stress suggests that although they did not find an aetiological connection between visual stress and dyslexia, their findings showed an interaction between the two conditions whereby “university students who experience high levels of visual stress are more likely to show improvements in reading rate with optimal colour if they also have dyslexia than if they do not have dyslexia” (p. 375). This suggests that although visual factors are not the cause of dyslexia, some interaction between dyslexia and visual functioning may be present. However, Wilkins (2003) summarized the research in the area and suggests that “the proportion of [dyslexic] children who benefit from overlays is similar to that in normal children” (p. 50). Furthermore, Handler and Fierson's (2011) more recent study summarized scientific literature on the topic and suggests that dyslexia and visual problems are unrelated:

Vision problems can interfere with the process of reading, but children with dyslexia or related learning disabilities have the same visual function and ocular health as children without such conditions. Currently, there is inadequate scientific evidence to support the view that subtle eye or visual problems cause or increase the severity of learning disabilities. (p. 818)

Therefore, research that has explored the connection between visual stress and dyslexia has been inconclusive, meaning that it would be misleading to think of dyslexia as a visual issue.

Therefore, Washburn et al. (2013) state that the teachers surveyed were misinformed about visual stress being directly related to dyslexia. Washburn et al. conclude that preservice teachers need to be taught up‐to‐date, evidence‐based information about the nature of dyslexia.

This study built on earlier research by Wadlington and Wadlington (2005) who conducted a study of 250 faculty members and students in a college of education in the United States and also found that inaccuracies were held about the direct relationship between visual issues and dyslexia. Both surveys asked teachers to use a Likert scale to indicate whether they thought a statement about dyslexics struggling with visual issues was true or false. As the teachers in these studies were prompted to consider the visual aspects of dyslexia, it is of interest to explore whether teachers mention the relationship between visual issues and dyslexia when they have not been promoted to do so.

Conclusions from these studies suggest that teachers' knowledge of dyslexia is not consistent and is mainly based on behavioural definitions. Furthermore, teachers appear to hold possible inaccuracies about dyslexia. However, a relatively small number of participants were used in these studies, and although they investigated how the teachers define and understand dyslexia, they did not investigate what impacts teachers' understanding. The present study will address this by surveying a larger sample and exploring the relationship between understanding of dyslexia and teacher training experiences.

## 2 PRESENT STUDY

The present study aims to investigate how teachers describe dyslexia, how the training teachers have received on dyslexia, and how this has impacted their knowledge and practice working with students with dyslexia.

### 2.1 Survey

The study was operationalized using an online questionnaire. The questionnaire contained a mixture of short answer questions and semantic differential scales. It was initially piloted on 56 teachers, followed by respondent debriefing with five teachers. Items were changed and amended before a final version was sent out via email.

In order to understand how teachers describe dyslexia, they were asked to “provide a short description of what [they] think dyslexia is.” Following this, participants were asked about their training on dyslexia. They were asked how well dyslexia was covered on their teacher training programme and whether they had received any additional training on dyslexia. They were also asked how confident they feel in helping a student with dyslexia achieve success.

### 2.2 Participants

Participants were recruited by emailing schools in England and in Wales in June 2016 and asking them to distribute the link to their teaching staff. A total of 4,314 teachers responded to the email, and approximately 2,900 completed the whole survey. Emailing all schools in England and Wales allowed for a good cross section of the population to be surveyed and large numbers for data analysis. The target population was classroom teachers in primary, secondary, further education, and special schools, in England and Wales. Respondents that did not fall within this population (determined by how demographic questions were responded to) were removed from data prior to analysis. This meant that on average, 2,570 teachers in the target population completed the survey (average due to item nonresponse).

Population figures of teaching staff were obtained from the respective Departments for Education in England and Wales. The data were weighted on setting, sex, and country. It should be noted that the data could not be weighted by other demographic variables (such as teacher type and years teaching) as this population information was not available from the Departments of Education. Therefore, although the weighted data give a more accurate reflection of the population, it does not account for all factors.

### 2.3 Coding

The descriptions were then coded using Frith's (1999) causal model in which she suggests that dyslexia can be described at three separate levels—biological, cognitive, and behavioural. The same coding method was applied by Bell et al. (2011). This suggests that it is an operational coding system to use when coding definitions of dyslexia. Descriptions that were coded as biological gave descriptors about the brain, neurological differences, or genetics being the cause of the dyslexic symptoms. Descriptions were coded as cognitive if they mentioned the cognitive processes associated with dyslexia, such as processing differences, issues decoding, and memory problems. Finally, descriptions that were coded as behavioural mentioned the outward symptoms of dyslexia, mainly issues with reading, writing, and spelling. If the participants mentioned more than one of these factors in their description, they were coded as having a combination.

Furthermore, DeVaus (2002) suggests that when coding, the researcher should first look for broad groupings and themes in the first 50 to 100 responses. From conducting this procedure, the responses seemed to show a theme that did not fit within the framework set out by Frith (1995). This was that many teachers were mentioning the visual stress aspects associated with dyslexia. As this appeared to be a key theme and related to previous research on teachers' understanding of dyslexia (Wadlington & Wadlington, 2005; Washburn et al., 2013), responses were also coded if they discussed visual aspects such as words moving round on the page or struggling to read black text on a white background.

### 2.4 Analysis

First, univariate analysis was conducted on all questions. This allowed for basic familiarization with the data and to understand the number of respondents that fell within certain categories. Bivariate analysis was then conducted using chi‐square tests (χ^2^). χ^2^ tests compare whether there is a significant difference between the expected value and the observed value in each subcategory or “cell.” However, although the overall χ^2^ result can tell us that there is a significant relationship between the variables, it cannot tell us which categories within the variable are driving the significant result. Therefore, adjusted standardized residuals can be calculated which identify which cells are making a significant contribution to the result. Cells that have adjusted standardized residuals that fall above or below ±1.96 are making a contribution to the significant chi‐square result.

## 3 RESULTS

### 3.1 Descriptors

Table 1 shows the number of respondents who provided each type of descriptor. The most mentioned descriptions were behavioural desperations, followed by participants mentioning a combination of both cognitive and behavioural descriptors.

**Table 1:** Frequency and percentage of descriptors provided

Description code	*N*	%
Biological	85	3.4
Cognitive	337	13.6
Behavioural	1,304	38.6
Biological and cognitive	14	0.6
Biological and behavioural	49	2.0
Cognitive and behavioural	551	22.2
Biological, cognitive, and behavioural	74	3.0
Does not exist	2	0.1
Other	71	2.8
Total	2,487	100

The responses were then recoded in order to determine the total number of participants who mentioned or did not mention each type of descriptor. A large majority of the respondents (79.5%) mentioned behavioural descriptors, followed by cognitive descriptors (39.3%). Biological descriptions were the most uncommon (9%).

Furthermore, it was also noted separately if the participant mentioned the visual factors associated with dyslexia. Four hundred twenty descriptions mentioned visual factors. This was 16.8% of the descriptions.

### 3.2 Teacher training

#### 3.2.1 Quality of teacher training

Teachers were asked “In your opinion how well was dyslexia covered on your teacher training programme?” A large majority of respondents (71.8%) said that dyslexia was not covered well at all on their teacher training programme.

#### 3.2.2 Additional training

Respondents were also asked if they had received any additional training on top of their ITT; the majority of teachers (50.4%) reported that they had no additional training on dyslexia.

#### 3.2.3 Impact of training

It was then interesting to investigate how training influenced whether or not a biological, cognitive, behavioural, or visual description of dyslexia was given when the respondents were asked to provide a description of dyslexia. Furthermore, the impact of training on the respondents' confidence working with the dyslexic students and the impact of years teaching were also investigated. Table 2 shows the cross‐tabulations and chi‐square statistics for these relationships.

**Table 2:** Chi‐square cross‐tabulations of relevant variables

	How well was dyslexia covered on your ITT programme?			Have you received any additional training of dyslexia since your ITT?			Years teaching				
	Not covered well	Covered well	χ^2^	No additional training	Additional training	χ^2^	Currently Training	NQT–5 years	5–10 years	10+ years	χ^2^
Biological not mentioned	1,966 (2.8)	141 (−2.8)	7.8**	1,176 (−0.4)	955 (0.4)	0.19	48 (−1.4)	471 (1.5)	424 (0.3)	1,317 (−1.0)	4.15
Biological mentioned	185 (−2.8)	25 (2.8)		122 (0.4)	93 (−0.4)		8 (1.4)	37 (−1.5)	40 (−0.3)	137 (1.0)	
Cognitive not mentioned	1,307 (0.4)	98 (−0.4)	0.19	867 (6.9)	552 (−6.9)	48.03**	40 (1.7)	340 (3.2)	287 (0.6)	840 (−3.6)	16.45**
Cognitive mentioned	844 (−0.4)	68 (0.4)		431 (−6.9)	495 (6.9)		16 (−1.7)	168 (−3.2)	177 (−0.6)	614 (3.6)	
Behavioural not mentioned	433 (−0.1)	34 (0.1)	0.01	244 (−1.9)	231 (1.9)	3.78	9 (−0.8)	87 (−2.1)	100 (0.7)	311 (1.4)	5.27
Behavioural mentioned	1,718 (0.1)	132 (−0.1)		1,054 (1.9)	817 (−1.9)		47 (0.8)	421 (2.1)	364 (−0.7)	1,142 (−1.4)	
Visual not mentioned	1,803 (2.1)	128 (−2.1)	4.37*	1,050 (−3.7)	906 (3.7)	13.63**	48 (0.5)	397 (−3.7)	375 (−1.7)	1,254 (4.3)	21.38**
Visual mentioned	358 (−2.1)	38 (2.1)		255 (3.7)	145 (−3.7)		8 (−0.5)	114 (3.7)	91 (1.7)	206 (−4.3)	
Confident	1,685 (−5.2)	162 (5.2)	26.67**	882 (−13.3)	979 (13.3)	176.7**	41 (−2.6)	452 (−6.5)	486 (−0.6)	1,596 (6.5)	57.57**
Unconfident	549 (5.2)	13 (−5.2)		454 (13.3)	121 (−13.3)		23 (2.6)	211 (6.5)	148 (0.6)	363 (−6.5)	

*Note*. Adjusted standardized residuals appear in parentheses beside group frequencies. NQT: newly qualified teacher.

* *p* < 0.05.

** *p* < 0.01.

#### 3.2.4 How well was dyslexia covered on ITT

Those who said dyslexia was covered well on the ITT were more likely to use a biological or visual descriptor when describing dyslexia. However, there was no significant effect of how well dyslexia was covered in ITT and whether the respondents gave a cognitive or behavioural description of dyslexia.

#### 3.2.5 Additional training

Teachers were also asked if they had received any additional training since their ITT. There was no significant effect of receiving any additional training and whether the respondents mentioned the biological or behavioural aspect associated with dyslexia. However, those that had received extra training were significantly more likely to use a cognitive descriptor and were significantly less likely to mention the visual aspects associated with dyslexia.

#### 3.2.6 Confidence about dyslexia

There was a significant effect of how teachers answered the question “how confident do you feel in helping a dyslexic person achieve success” on how teachers responded to the question “In your opinion how well was dyslexia covered on your initial teacher training programme.” Those who felt confident were significantly more likely to say it was covered well than those that felt unconfident. Furthermore, there was a significant effect of confidence, on whether the respondents had received any additional training on dyslexia. Those that were confident were significantly more likely to have received additional training than those that were unconfident.

#### 3.2.7 Years teaching

Although there was no effect of years teaching on whether the respondent gave a biological or behavioural descriptor of dyslexia, there was a significant effect of the number of years teaching on whether a cognitive or visual descriptor was given. Those that had been teaching from their newly qualified teacher (NQT) year to 5 years were less likely to use a cognitive descriptor, whereas those teaching for more than 10 years were more likely to use a cognitive descriptor. In contrast, those that had been teaching from the NQT year to 5 years were more likely to mention visual factors and those that had been teaching for more than 10 years were less likely to use visual factors.

Additionally, years teaching had a significant effect on confidence whereby those teaching for 5 years and under were more likely to feel unconfident in helping a dyslexic person achieve success, whereas those who had been teaching for more than 10 years stated they were more confident.

## 4 DISCUSSION

First, from looking at the descriptions that teachers gave of dyslexia, it is clear that most teachers understand dyslexia in terms of how it affects pupils at the behavioural level. This supports findings from other research that has also shown that both the lay public and teachers use behavioural descriptors when thinking about dyslexia (Bell et al., 2011; Furnham, 2013; Mortimore, 2013; Washburn et al., 2013). It could be hypothesized that this is because teachers are more likely to witness the behavioural correlates of dyslexia in the classroom. However, as Frith (1995) suggests, it is important to understand all three levels of dyslexia. If teachers simply think of dyslexia as something that affects “reading, writing and spelling,” they may make assumptions about the pupils expected performance in these areas. This concurs with a “stereotypical” view of dyslexia. It would be more useful to think of dyslexia using all three levels of Frith's model. In particular, it is useful for teachers to understand dyslexia at the cognitive level as the “weaker” cognitive functions can be developed through effective teaching practice.

It was also noted that 16.8% of teachers mentioned visual factors. Therefore, nearly twice as many teachers mentioned visual factors than biological factors. This also supports findings from previous research that suggests teachers hold the understanding that dyslexia and visual functioning are related (Wadlington & Wadlington, 2005; Washburn et al., 2013). The current survey demonstrates that teachers mention visual issues when describing dyslexia, despite research being inconclusive about this relationship.

However, it is important to state that the blame here should not lie with the teachers, but rather with the education institution for not ensuring that teachers are entering the workforce with adequate knowledge of how to best help a dyslexic student. A large majority of teachers said that dyslexia was “not covered well at all” on their initial teacher education programme. Furthermore, it could, perhaps, be expected that those who had recently finished their teacher training would hold more up‐to‐date, evidenced‐based knowledge on dyslexia. However, those who had been teaching from NQT to 5 years were significantly less likely to use a cognitive descriptor. As the newer teachers will have completed their ITT more recently, this suggests that current teacher training is not including sufficient detail on the processing issues that are known to be associated with dyslexia. As previously mentioned, the most effective interventions focus on improving cognitive processing (Rose, 2009; Snowling & Hulme, 2011). Consequentially, it is vital that teachers are aware of this so that they can help their students most effectively. For teachers to be aware of this, information about cognitive processing must be included in any training that teachers receive about dyslexia during ITT; the results suggest that this is not currently the case. Additionally, those who had been teaching from the NQT to five years were more likely to mention visual factors, whereas those who had been teaching for 10 or more years were less likely to mention visual factors. Therefore, it appears that more recent teacher training is teaching that dyslexia is associated with visual processing despite evidence being unable to show a direct link between dyslexia and visual factors. In contrast, those that had been in the teaching profession for longer were more likely to mention cognitive descriptors and were less likely to mention visual descriptors. It could be assumed that these teachers will have had more access to CPD and additional training on dyslexia over their teaching career, compared with newer teachers. This may have increased their knowledge of these aspects of dyslexia.

A key issue that stems from these findings is the lack of effective training given to teachers during their ITT. However, on a more positive note, the results show that extra training increases teachers' confidence helping students with dyslexia. Additionally, those who had extra training were more likely to use cognitive descriptors and were less likely to use visual descriptors. This suggests that extra training has a significant positive effect on teachers work with dyslexic pupils.

### 4.1 Implications for policy and practice

The results presented in this paper have implications for teacher training in England and in Wales. Although the National Teaching Standards state that teachers must be able to engage with students with all needs (Department for Education, 2011), a large majority of teachers claimed that dyslexia was “not covered well at all” on their ITT programme, suggesting that they are ill‐equipped to meet this requirement when entering the workforce. Therefore, an initial recommendation is for compulsory teaching of dyslexia on ITT courses in England and in Wales. This training should be evidence based, using up‐to‐date academic knowledge, which covers the biological, cognitive, and behavioural aspects of dyslexia. Of particular importance is to provide knowledge of the cognitive aspects of dyslexia, such as phonological processing, which is known to inform the most effective interventions.

Second, as suggested by the Carter and Tabberer reviews on teacher training, CPD is needed in order to increase the knowledge of teachers currently in the workforce (Department for Education, 2015; Department for Education and Skills, 2013). The results highlight that extra training can have a significant positive effect; therefore, this paper calls for a continuation and increase in the delivering of CPD on dyslexia. This training should be provided at regular intervals during a teacher's career to ensure that they are aware of the most up‐to‐date information and research on dyslexia.

### 4.2 Limitations of the study

As participation in the survey was voluntary, teachers that responded could be deemed as more engaged with the subject of dyslexia than others. Consequentially, this could cause potential bias in the sample. However, a large breadth of teachers with differing knowledge and experience were surveyed; therefore, it does not appear to be biased to a particular type of teacher. Furthermore, by weighting the data, the teacher population demographics of gender, school type, and country were accounted for in the sample.

Another limitation of the current study is that it does not acknowledge the methods that teachers use when working with students with dyslexia. Therefore, although we can assume that poor knowledge leads to poor practice, this cannot be discerned from this study. Nevertheless, it is significant that the teachers surveyed lacked the knowledge of the cognitive aspects of dyslexia that have been shown to be important in effective interventions for those with dyslexic symptoms. Future research should investigate how a teacher's knowledge of dyslexia influences their practice. If it is found that poor knowledge of dyslexia leads to poor practice, this strengthens the argument for more thorough coverage of dyslexia during ITT and CPD.

## 5 CONCLUSION

The current study suggests that a majority of teachers base their understanding of dyslexia on behavioural level descriptors, mainly that individuals with dyslexia struggle with reading, writing, and spelling. However, as Frith (1995) points out, understanding of the biological and cognitive aspects of dyslexia is also important for a good understanding of dyslexia. Results from both the current and previous research show that biological and cognitive factors are not as commonly mentioned by teachers. Furthermore, the understanding that dyslexia is a visual issue is still a prevailing discourse when teachers describe dyslexia. The lack of understanding of the underlying behavioural and cognitive difficulties associated with dyslexia, and the inaccuracies held, may be due to the fact that the teachers surveyed said that dyslexia was “not covered well at all” on their ITT programme. A good understanding of dyslexia is important in successfully intervening to best help those with dyslexia. Therefore, findings from this research show that good‐quality, evidenced‐based training is essential so that teachers have a better understanding of the multilayered aspects of dyslexia and to dispel any inaccuracies they hold.
